# The anti-myeloma activity of bone morphogenetic protein 2 predominantly relies on the induction of growth arrest and is apoptosis-independent

**DOI:** 10.1371/journal.pone.0185720

**Published:** 2017-10-13

**Authors:** Charlotte Lagler, Mohamed El-Mesery, Alexander Christian Kübler, Urs Dietmar Achim Müller-Richter, Thorsten Stühmer, Joachim Nickel, Thomas Dieter Müller, Harald Wajant, Axel Seher

**Affiliations:** 1 Department of Oral and Maxillofacial Plastic Surgery, University Hospital Wuerzburg, Wuerzburg, Germany; 2 Department of Biochemistry, Faculty of Pharmacy, Mansoura University, Mansoura, Egypt; 3 Comprehensive Cancer Center Mainfranken (CCCMF), University Hospital of Wuerzburg, Wuerzburg, Germany; 4 Chair Tissue Engineering and Regenerative Medicine, University Hospital Wuerzburg, Wuerzburg, Germany; 5 Fraunhofer IGB, Translational Center Wuerzburg "Regenerative therapies in oncology and musculoskeletal diseases", Wuerzburg, Germany; 6 Julius-von-Sachs-Institute, Department of Molecular Plant Physiology and Biophysics, University of Wuerzburg, Wuerzburg, Germany; 7 Division of Molecular Internal Medicine, Department of Internal Medicine II, University Hospital Wuerzburg, Wuerzburg, Germany; Institute of Biochemistry and Biotechnology, TAIWAN

## Abstract

Multiple myeloma (MM), a malignancy of the bone marrow, is characterized by a pathological increase in antibody-producing plasma cells and an increase in immunoglobulins (plasmacytosis). In recent years, bone morphogenetic proteins (BMPs) have been reported to be activators of apoptotic cell death in neoplastic B cells in MM. Here, we use bone morphogenetic protein 2 (BMP2) to show that the "apoptotic" effect of BMPs on human neoplastic B cells is dominated by anti-proliferative activities and cell cycle arrest and is apoptosis-independent. The anti-proliferative effect of BMP2 was analysed in the human cell lines KMS12-BM and L363 using WST-1 and a Coulter counter and was confirmed using CytoTox assays with established inhibitors of programmed cell death (zVAD-fmk and necrostatin-1). Furthermore, apoptotic activity was compared in both cell lines employing western blot analysis for caspase 3 and 8 in cells treated with BMP2 and FasL. Additionally, expression profiles of marker genes of different cell death pathways were analysed in both cell lines after stimulation with BMP2 for 48h using an RT-PCR-based array. In our experiments we observed that there was rather no reduction in absolute cell number, but cells stopped proliferating following treatment with BMP2 instead. The time frame (48–72 h) after BMP2 treatment at which a reduction in cell number is detectable is too long to indicate a directly BMP2-triggered apoptosis. Moreover, in comparison to robust apoptosis induced by the approved apoptotic factor FasL, BMP2 only marginally induced cell death. Consistently, neither the known inhibitor of apoptotic cell death zVAD-fmk nor the necroptosis inhibitor necrostatin-1 was able to rescue myeloma cell growth in the presence of BMP2.

## Introduction

Multiple myeloma (MM) is a malignant disease and is a B-cell lymphoma. It is characterized by the monoclonal proliferation of plasmatic cells in the bone marrow leading to an increase in immunoglobulins (plasmacytosis) [1]. MM typically leads to enhanced susceptibility to infections and organ damage, and it may involve massive destruction of bone structures (osteolysis) [2]. Approximately 10% of all haematological cancers and 1% of all cancers are MM [3]. The exact origin of the disease remains unknown, and it is assumed that several different genetic factors contribute to the MM pathology [4, 5].

In the past, several studies have suggested that bone morphogenetic proteins (BMPs) induce apoptosis in MM cells. BMPs are members of the TGF-beta superfamily, which consists of more than 30 growth factors, the most prominent representatives of which are the eponymous TGF-betas. The BMPs form a functionally important subgroup of this family and possess a high osteo-inductive potential. Classically, these factors have been shown to play significant roles in bone development, as well as bone homeostasis and regeneration, but they have also been implicated in the regulation of other important biological processes, such as embryogenesis and organogenesis [6–8].

The first ligand of the TGF-beta superfamily demonstrated to have apoptotic potential was Activin A in 1993 [9]. Zipori *et al*. described the general role of Activin A in the negative regulation of normal and neoplastic B cells [10]. In 2000, BMPs, specifically BMP2, were found to induce apoptosis in MM cells [11]. BMP4, BMP5, BMP6, BMP7 and BMP9 were subsequently reported to also trigger apoptosis in MM cells [12–17].

Apoptosis is a complex biochemically stringently regulated process that involves controlled cell demise. In addition to its general role in the regulation of the immune system (e.g., the negative selection of lymphocytes [18]) and organogenesis during embryonic development (e.g., of the limbs [19]), apoptosis is essential for permanent elimination of potentially degenerated or already malignant cells [20–22]. Apoptosis is characterized by the two following decisive conditions: speed and efficiency. Cell death is initiated as soon as cell- or cell cluster (tissue) elimination becomes inevitable, as otherwise these cells represent an existential threat. For this reason, apoptotic cells are completely dissolved within hours except for few remaining apoptotic bodies (apobodies). To ensure high speed and efficiency of apoptosis even under unfavourable conditions, no *de novo* synthesis of RNA or proteins is necessary for apoptosis because the entire apoptosis framework is readily available [23–26].

In this study, we show that the assumed “apoptotic” effect of BMP2 on human MM cells is limited and outcompeted by an anti-proliferative and/or cell cycle-arresting effect. Thus, in MM, BMP2-induced apoptosis presents a rather indirect side-effect that is neither quantitatively nor qualitatively comparable to that of an approved apoptotic factor, such as FasL.

## Methods

### Preparation of the ligands BMP2, Fc-FLAG-FasL and FLAG-TNF-alpha

A cDNA fragment encoding amino acid residues 283–396 of BMP2 plus an N-terminal extension (Met-Ala) was cloned into a bacterial expression vector [27]. BMP2 was expressed in *E*. *coli*, recovered from inclusion bodies, refolded, and purified as previously described [28]. Affinity chromatography with anti-FLAG M2 agarose beads (Sigma-Aldrich) was used to purify human recombinant FLAG-tagged soluble Fc-FLAG-FasL and FLAG-TNF-alpha from the supernatants of HEK293 cells that were stably transfected with a corresponding expression plasmid.

### Cell culture

The human MM cell lines KMS12-BM and L363 and the murine cell line C2C12 were obtained from the German Collection of Microorganisms and Cell Cultures (DSZM; Germany). The murine MM cell line MPC11 and Jurkat A3 cells were obtained from the ATCC collection (LGC Standards GmbH, Germany). KMS12-BM, L363 and Jurkat A3 cells were maintained in RPMI 1640 (Gibco, Life Technologies, Germany) supplemented with 10% fetal calf serum (FCS) (Biochrom, Berlin, Germany), 100 U/ml penicillin, 100 μg/ml streptomycin (PAN Biotech, Aidenbach, Germany), 2 mM glutamine (PAA, Pasching, Austria), and 1 mM Na-pyruvate (PAN Biotech, Germany). C2C12 cells were cultured in Dulbecco’s modified Eagle’s medium (DMEM; with 10% FCS, 100 U/ml penicillin, 100 μg/ml streptomycin), and MPC11 cells were cultured in RPMI (with 10% FCS, 100 U/ml penicillin, 100 μg/ml streptomycin). All cells were grown at 37°C in 5% CO_2_.

### Primary MM cells/CD138^+^ selection

Bone marrow aspirates from MM patients were obtained at the Universitaetsklinikum Wuerzburg, Medizinische Klinik und Poliklinik II, after written informed consent, within the frame of sample acquisition for the Clinical Research Unit 216 (KFO216) "Multiple Myeloma". Permission was granted by the local ethics committee (Ethik-Kommission der Medizinischen Fakultaet der Universitaet Wuerzburg; reference number 76/13). BM aspirates were subjected to density centrifugation (Lymphocyte Separation Medium; PAA). The mononuclear cell fraction was rinsed with phosphate-buffered saline (PBS) and cold separation buffer (PBS containing 0.5% FCS and 2.5 mM EDTA), incubated with CD138 microbeads (Miltenyi Biotech, Bergisch Gladbach, Germany), and subjected to MACS Large Cell Columns (Miltenyi Biotech). The columns were thoroughly washed with separation buffer. The myeloma cells were eluted from the column and seeded onto bone marrow stromal cells (BMSCs) or cultured in medium supplemented with human IL-6 (2 ng /ml) (R&D, Germany). The cells were seeded at a density of 30,000 cells/well in 96-well plates, and BMP2 (250 nM) was added. The cells were subsequently cultured for 3 days prior to performing WST-1 assays, and the final values were calculated relative to those in the untreated control cells. Each assay was performed in duplicate unless low MM cell numbers permitted only single measurements to be obtained.

### Coulter counter assay

The cell numbers of the cell lines KMS12-BM and L363 were determined using a Coulter counter CASY^®^. Then, 2 x 10^4^ cells were seeded at 100 μl/per well in 96-well plates and stimulated with 250 nM BMP2 for 24 h, 48 h, 72 h and 96 h. For control a set of cells remained non-stimulated. At indicated time points, cell numbers were determined using CASY^®^. Cells that were smaller than 8 micrometre were considered dead. All experiments were performed with technical duplicates and repeated three times.

### WST-1 assay

The cell numbers of the cell lines KMS12-BM and L363 were determined using the Coulter counter CASY^®^. Then, 2 x 10^4^ cells were seeded at 100 μl/per well in 96-well plates and stimulated with 250 nM BMP2 for 24 h, 48 h, 72 h und 96 h. For control a set of cells remained non-stimulated. At indicated time points, WST-1 (10 μl/well) (Roche, Germany) was added, and the cells were incubated for exactly 3 h at 37°C to standardize the results across the different time points. The intensity of WST-1 staining was measured at 450 nm using a micro plate reader (TECAN RAINBOW^®^, Germany). All assays were performed in duplicate, and the experiments were repeated three times.

### BMP2-induced inhibition of MPC11 cell proliferation

Murine myeloma cell line MPC11 cells were seeded in DMEM in 96-well plates at a density of 5 x 10^3^ cells / well. Dose-dependent inhibition of proliferation was measured by adding increasing concentrations of BMP2. After 72 h, 10 μl of [3H]-thymidine (0.25 μCi; GE Healthcare/Amersham, Munich, Germany) was added to each well. After 24 h cells were immobilized on fibre mats (Skatron Instruments A/S, Lier, Norway), and thymidine incorporation was analysed using an RITA counter (Raytest, Straubenhardt, Germany). All assays were performed with technical duplicates, and the experiments were repeated three times.

### C2C12 alkaline phosphatase assay (ALP assay)

Murine C2C12 cells were seeded at a density of 1.2 × 10^4^ cells / well in 96-well micro titre plates and stimulated for 3 days with 0.5–250 nM BMP2 in 100 μl of DMEM containing 2% FCS and antibiotics (100 U /ml penicillin and 100 μg/ml streptomycin) at 37°C and 5% CO_2_. After 72 h, cells were washed with PBS and lysed for 1 h by adding 100 μl of 1% NP-40 in ALP buffer (0.1 M glycine, pH 9.6, 1 mM MgCl_2_, and 1 mM ZnCl_2_). ALP activity was determined by incubating the cell lysate for 15 min with 100 μl of ALP buffer supplemented with 1 mg/ml *p*-nitrophenylphosphate (Sigma Aldrich, Germany) and measuring absorbance at 405 nm using a micro plate reader (TECAN RAINBOW, Germany). All assays were performed in duplicate, and the experiments were repeated three times.

### CytoTox assay

Jurkat A3 cells (2x10^4^/well) were grown overnight in 100 μl of culture medium in 96-well plates. The following day, the cells (measurements were done in triplicate) were pre-incubated for 1 h with the NEDD8 activating enzyme (NEA) inhibitor MLN4924 (MLN; 20 μM; Active Biochemicals Co., Hong Kong, China), the pan-caspase inhibitor benzyloxycarbonyl-Val-Ala-Asp (OMe) fluoromethylketone (zVAD-fmk) (50 μM; Bachem AG, Weil am Rhein, Germany) and the necroptosis inhibitor necrostatin-1 (nec-1; 90 μM; Enzo Life Sciences, Loerrach, Germany). The cells were then incubated overnight with TNFα (100 ng /ml). As necrostatin-1 will cross-react with WST-1, Jurkat A3 cell viability was measured using the 3-(4,5-dimethylthiazol-2-yl)-2,5-diphenyltetrazolium bromide (MTT) assay. Results were quantified by measuring absorbance at 570 nm using a micro plate reader (TECAN RAINBOW, Germany).

### Western blot analysis

For Western blot analysis of whole cell lysates, cells were scraped into ice-cold PBS, collected via centrifugation, sonicated (10 pulses) and then lysed by boiling (5 min at 96°C) in 4x Laemmli sample buffer (8% SDS, 0.1 M DTT, 40% glycerol, and 0.2 M Tris-HCl pH 8.0). Proteins were separated by SDS-PAGE and transferred onto nitrocellulose membranes. After nonspecific binding was blocked by incubating the membranes in TBS containing 0.1% Tween 20 and 5% dry milk, immunoblotting was performed with primary antibodies specific for proteolytically cleaved Caspase 3 (Cell Signaling Technology, Danvers, MA, USA), Caspase 8 p18 (Santa Cruz Biotechnology, Heidelberg) and the respective HRP-conjugated secondary antibodies (Dako, Hamburg, Germany). Detection was performed by enhanced chemoluminescence using ECL Plus reagents (Amersham-Pharmacia, Freiburg, Germany). Experiments were repeated three times.

### Analysis of RNA expression profiles using qiagen RT^2^ RT-PCR arrays

A total of 1 x 10^6^ cells were incubated with or without 250 nM BMP2 for 48 h, and RNA was subsequently isolated using the Qiagen RNeasy Mini Kit (Qiagen, Germany) according to the manufacturer’s recommendations. cDNA was synthesized using the RT^2^ first-strand kit (Qiagen) as indicated in the manual. The cDNA was subsequently analysed using RT^2^ SYBR Green qPCR master mix and the RT^2^ Profiler PCR Array PAHS-212Z “Human Cell Death Pathway Finder” (Qiagen) and a Bio-Rad CFX96 PCR cycler following the manufacturer’s instructions. The following thermal profile was used: 1 cycle at 95°C and 40 cycles at 95°C for 15 s followed by 60°C for 1 min.

Raw data were analysed using Qiagen online software (http://pcrdataanalysis.sabiosciences.com/pcr/arrayanalysis.php). The RT^2^ Profiler PCR Array data analysis software was used to calculate fold-changes based on the widely approved ΔΔC_t_ method. For normalization, the expression of all genes was used. C_t_ values >35 were defined as no expression.

The “Human Cell Death pathway finder” contained the following genes:

#### Apoptosis

Pro-Apoptotic: ABL1, APAF1, ATP6V1G2, BAX, BCL2L11, BIRC2 (c-IAP1), CASP1 (ICE), CASP3, CASP6, CASP7, CASP9, CD40 (TNFRSF5), CD40LG (TNFSF5), CFLAR (Casper), CYLD, DFFA, FAS (TNFRSF6), FASLG (TNFSF6), GADD45A, NOL3, SPATA2, SYCP2, TNF, TNFRSF1A (TNFR1), TNFRSF10A (TRAIL-R), and TP53 (p53). Anti-Apoptotic: AKT1, BCL2, BCL2A1 (BFL1), BCL2L1 (BCLXL), BIRC3 (c-IAP2), CASP2, IGF1R, MCL1, TNFRSF11B (OPG), TRAF2, and XIAP (BIRC4).

#### Autophagy

AKT1, APP, ATG12, ATG16L1, ATG3, ATG5, ATG7, BAX, BCL2, BCL2L1 (BCLXL), BECN1, CASP3, CTSB, CTSS, ESR1 (ERα), FAS (TNFRSF6), GAA, HTT, IFNG, IGF1, INS, IRGM, MAP1LC3A, MAPK8 (JNK1), NFKB1, PIK3C3 (Vps34), RPS6KB1, SNCA, SQSTM1, TNF, TP53 (p53), and ULK1.

#### Necroptosis

ATP6V1G2, BMF, C1orf159, CCDC103, COMMD4, CYLD, DEFB1, DENND4A, DPYSL4, EIF5B, FOXI1, GALNT5, GRB2, HSPBAP1, JPH3, KCNIP1, MAG, OR10J3, PARP1 (ADPRT1), PARP2, PVR, RAB25, S100A7A, SPATA2, SYCP2, TMEM57, TNFRSF1A (TNFR1), and TXNL4B.

## Results

### BMP2 is biologically active in C2C12 and MPC-11 cells

**Fig 1:**
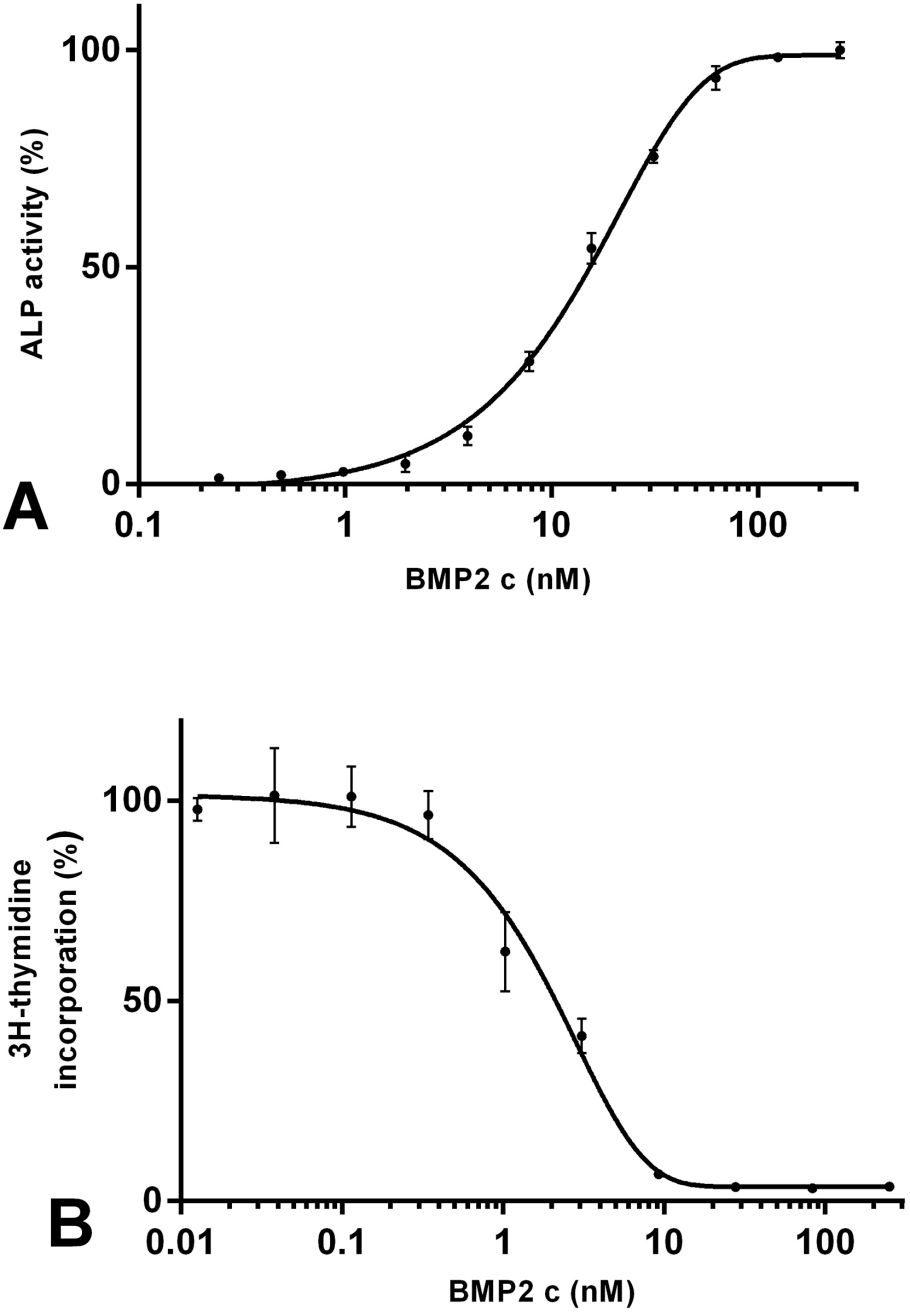
A+B. Biological activity of BMP2. The biological activity of BMP2 was evaluated in two well-established cell systems. (A) BMP2-induced ALP expression in murine C2C12 cells. (B) The anti-proliferative effect induced by BMP2 in murine MPC11 cells was determined from DNA synthesis as measured via 3H-thymidine incorporation.

In order to demonstrate the functionality of the BMP2 used in this study, biological activity was initially determined using two different well-established assays [29–32]. A characteristic function of BMP2 is the induction of bone growth. We therefore evaluated the BMP2-stimulated expression of the osteogenic marker alkaline phosphatase (ALP). Expression of ALP can be determined using a photometric assay (ALP assay). In accordance with results described in the literature, we obtained an EC50-value for BMP2-induced expression of ALP in C2C12 cells of 10–20 nM (Fig 1A) [29, 31, 32]. In addition, the inhibitory effect of BMP2 on the proliferation of the murine myeloma B cell line MPC-11 was determined by analysing incorporation of [3H]-thymidine into DNA [31]. In accordance with previously published results, we observed an IC50-value of 1–3 nM for BMP2-mediated inhibition of cell proliferation (Fig 1B) [30].

### BMP2 has an anti-proliferative effect in the human MM cell lines KMS12-BM und L363

**Fig 2:**
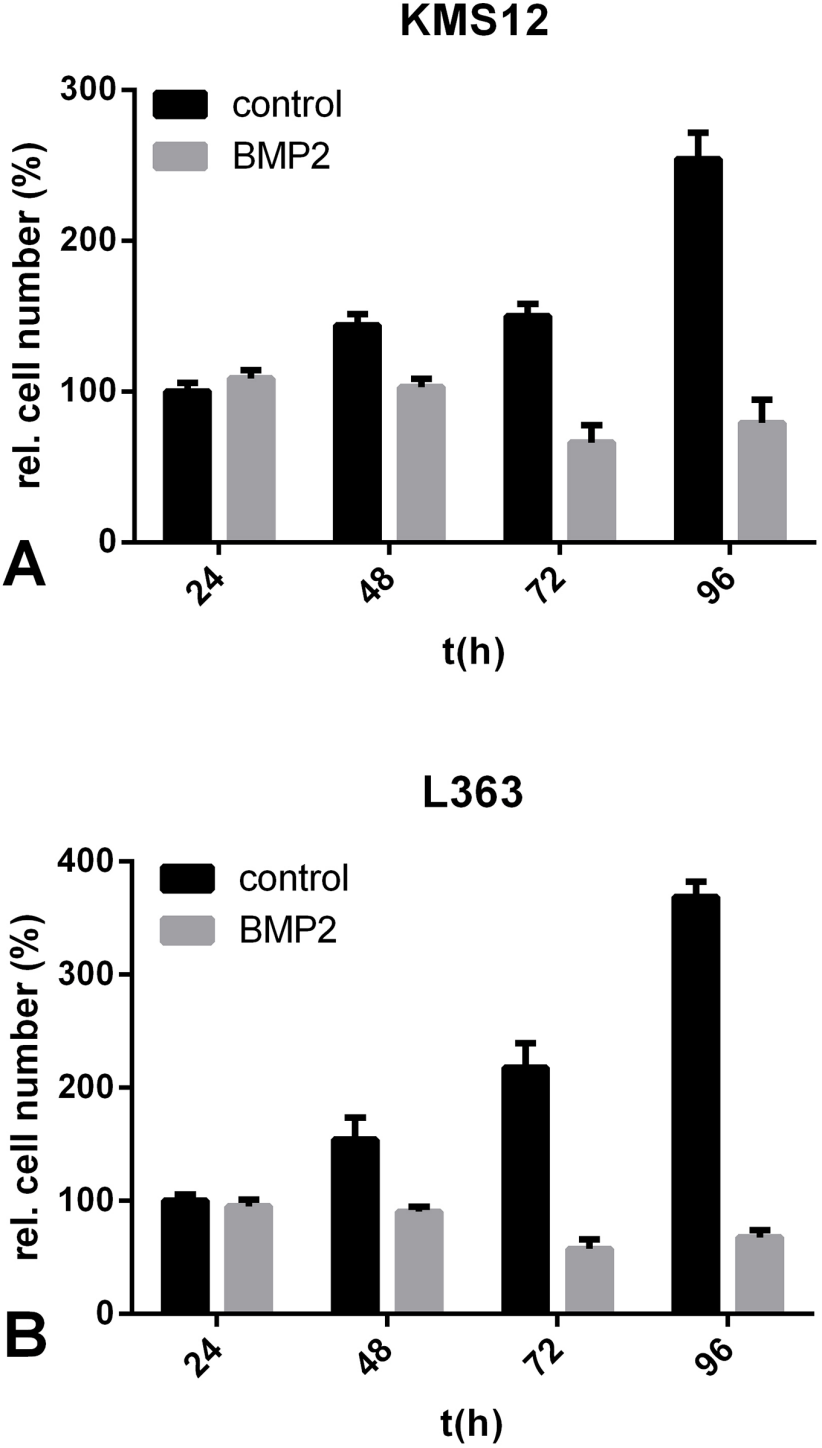
A+B. BMP2 induced an anti-proliferative effect in KMS12-BM und L363 cells. (A) KMS12-BM and (B) L363 cells were treated with or without 250 nM BMP2. Cell viability was determined after 24 h, 48 h, 72 h and 96 h by measuring NADH/H+ using the WST-1 assay. Relative cell numbers were normalized to 100% at time point zero.

For further analysis, we used the human MM cell lines KMS12-BM (previously published to be BMP2-sensitive) [33] and L363. The cells were stimulated with BMP2 for 24 h, 48 h, 72 h and 96 h, and metabolic activity was determined using the WST-1 assay. Cells in the untreated control groups proliferated continuously during the time frame. Noteworthy, BMP2-stimulated cells showed slightly lower rates of metabolic activity than those of the controls. After 96 h, metabolic activity dropped to 79% of baseline (compared to the beginning of the stimulation) in BMP2-treated KMS12-BM cells and to 67% in BMP2-treated L363 cells, whereas metabolic activity was increased in the untreated cells by 3.2-fold in the KMS12-BM and 5.4-fold in the L363 cells (compared to the time point at start). Hence, after 96 h, treatment with BMP2 resulted in 31% less cell viability in KMS12-BM cells and 18% less cell viability in L363 cells than was observed in the untreated control cells (Fig 2A and 2B).

### BMP2 did not decrease absolute cell number of KMS12-BM and L363

**Fig 3:**
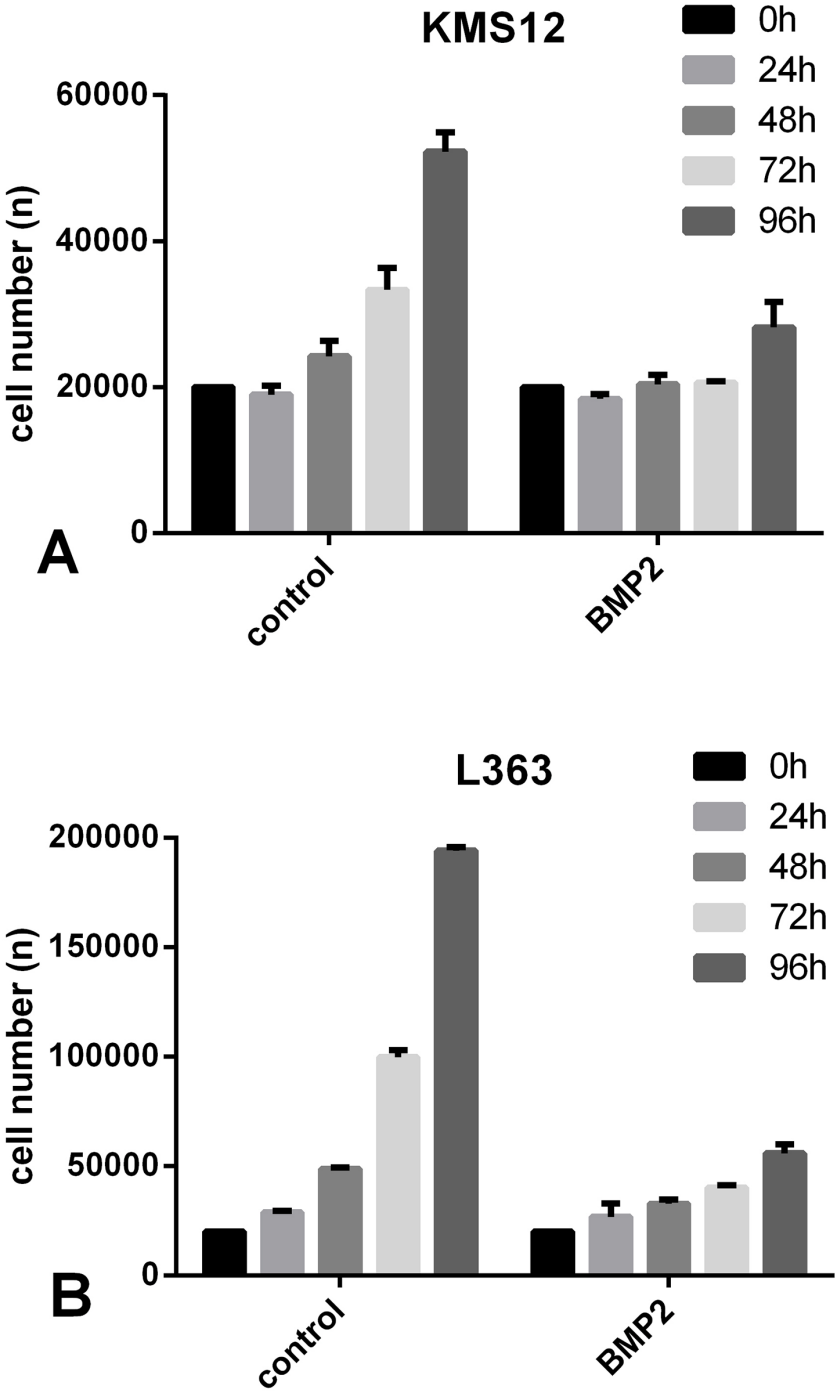
A+B. Absolute cell numbers were not reduced after stimulation with BMP2. (A) KMS12-BM and (B) L363 cells were treated with or without 250 nM BMP2. Absolute cell number (n) was measured after 0 h, 24 h, 48 h, 72 h and 96 h using a Coulter counter.

Because conversion of WST-1 measures only metabolic activity of the cells, it was not possible, particularly in experiments running several days, to determine whether the observed decrease in metabolic activity is due to induction of cell death, attenuated proliferation, attenuated metabolic activity or due to a combination of two or three of those effects. We therefore aimed to determine the effect of BMP2 on viability at the cellular level by directly counting cell numbers using a Coulter counter. The Coulter counter system determines cell diameter as well as the impedance of a single cell and from these two parameters it is not only possible to distinguish between viable and dead cells but also absolute cell numbers can be determined. Starting the experiment with 20,000 cells, the absolute cell numbers were measured after 24 h, 48 h, 72 h and 96 h. In both the KMS12-BM and the L363 cells, after 96 h, the number of cells was substantially lower in the BMP2-stimulated group compared to the untreated cell groups. However, we were surprised that during the 4 days of the experiment the absolute number of live cells in the groups treated with BMP2 did not decrease below the number of cells initially seeded (Fig 3A and 3B). This effect of BMP2 on the number of live cells may represent the induction of apoptosis in a few cells, but it might also be the result of BMP2-medited anti-proliferative activity or a combination of increased cell death and inhibition of proliferation“

### The BMP2-induced inhibition of cell division is not rescued by inhibitors of programmed cell death

**Fig 4:**
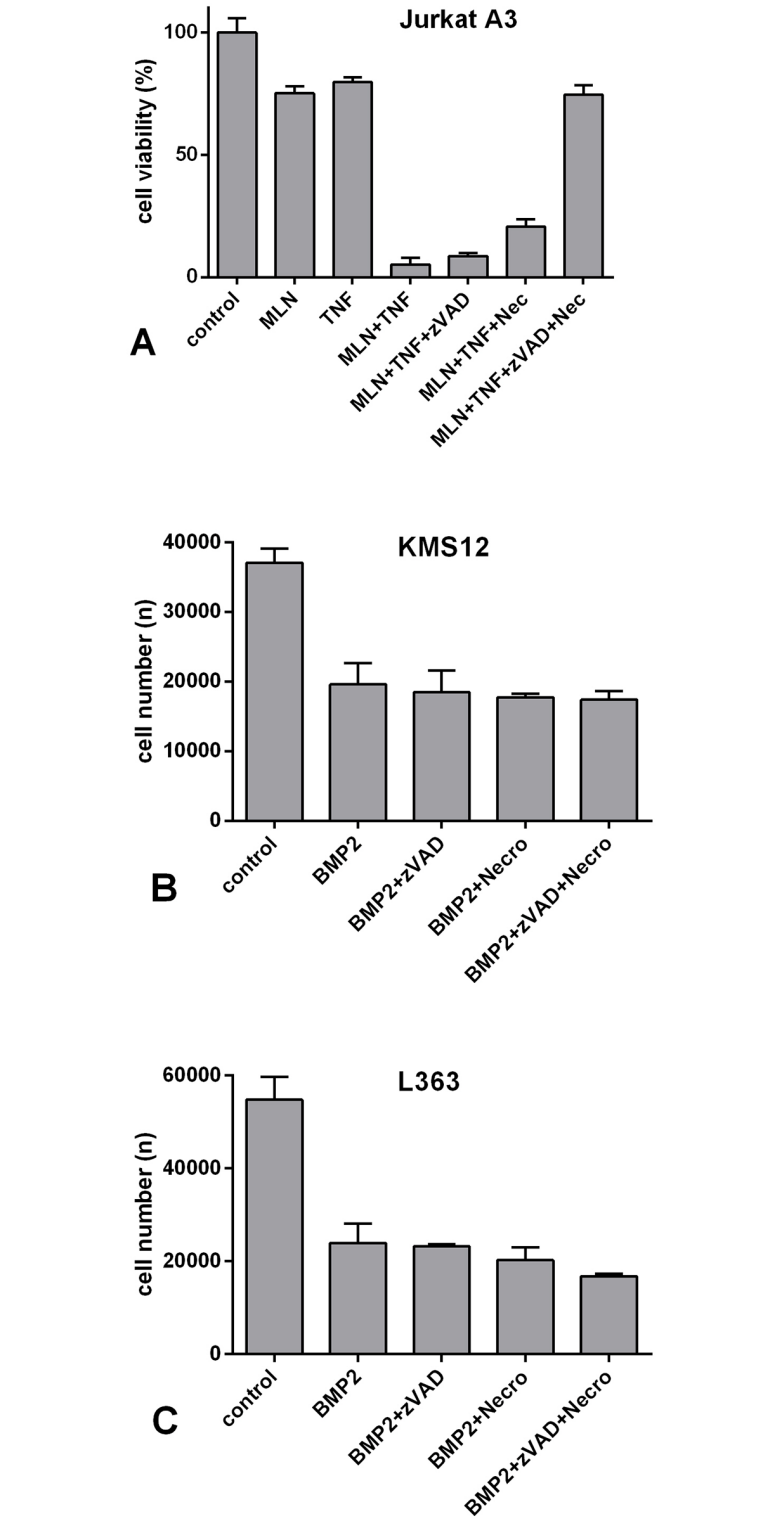
A-C. Inhibitors of cell death (zVAD-fmk and Nec-1) do not rescue the BMP2-induced decrease in cell numbers. (A) The inhibitors zVAD-fmk and Nec-1 were first tested to assess their biological activity. Jurkat A3 cells were grown overnight in 96-well plates. The following day, the cells were incubated with the inhibitors MLN (20 μM), zVAD-fmk (50 μM) and necrostatin-1 (90 μM) using the concentrations indicated. After 1 h, TNFalpha (100 ng/ml) was added, and the cells were incubated overnight. Cell viability was determined using CytoTox. (B) KMS12-BM and (C) L363 cells were incubated with the inhibitors MLN (20 μM), zVAD-fmk (50 μM) and necrostatin-1 (90 μM) at the concentrations indicated. After 1 h, BMP2 (250 nM) was added, and the cells were incubated for 96 h. Cells of the control group were left untreated. Cell number was determined by cell counting using a Coulter Counter.

Next, we wanted to determine whether programmed cell death contributed to the effect of BMP2. We therefore evaluated the impact of two inhibitors, zVAD-fmk and necrostatin-1, which specifically inhibit apoptosis and necroptosis, onto stimulation of the cells with BMP2. In general, apoptosis is activated via one of the following two distinct pathways: the intrinsic and the extrinsic pathway. Both pathways conclude in the proteolytic cleavage and activation of effector caspases, such as caspase 3 [34]. Necroptosis is a caspase-independent process that is typically initiated by receptor-interacting serine/threonine-protein kinase 1 (RIPK1). On a molecular level, apoptosis and necroptosis are closely linked [35–37]. When apoptosis is initiated, necroptosis is generally suppressed through the inhibition of RIPK1 by activated caspase 8 [38–40]. Cancer cells that express large amounts of anti-apoptotic proteins or cells infected with a virus often express non-activatable or inhibited caspase 8 [41], which impedes apoptosis in these cells. However, RIPK1, which initiates necroptosis as an emergency programme, accumulates in these cells [38–40, 42]. The compound zVAD-fmk irreversibly inhibits initiator as well as effector caspases thereby blocking apoptosis [43], while Nec-1 interferes with RIPK1 to specifically inhibit necroptosis [44]. For these experiments, it was important that both inhibitors are applied simultaneously as sole inhibition of caspase 8 by zVAD-fmk might activate necroptosis as a backup to the cell death pathway. Hence, simultaneous application of both inhibitors will completely shut down any programmed cell death. To validate the effectiveness of the two inhibitors we stimulated Jurkat A3 cells with TNF-alpha and the NEDD8 activating enzyme (NEA) inhibitor MLN4924, the latter of which inhibits NF-kappaB signalling and thereby sensitizes cells to TNF-induced cell death. In this control experiment the overnight incubation with both TNF-alpha and MLN4924 led to an almost loss of cellular viability, indicating nearly complete cell death [45, 46]. When applied alone, zVAD-fmk and Nec-1 rescued a fraction of the cells from cell death; only when applied in combination cells were completely protected from cell death (Fig 4A). This finding indicates that the Jurkat cells exhibit sensitivity to TNF-induced apoptosis as well as necroptosis and confirms the functionality of both zVAD-fmk and Nec-1.

Both inhibitors were therefore used in experiments involving BMP2-treated KMS12-BM and L363 cells. However, independent as to whether used alone or in combination, the two inhibitors showed no significant effect on the absolute number of viable cells at any of the examined time points (0–96 h), as determined by direct cell counting using a Coulter counter. Representative results obtained after 96 h are shown (Fig 4B and 4C).

**Fig 5:**
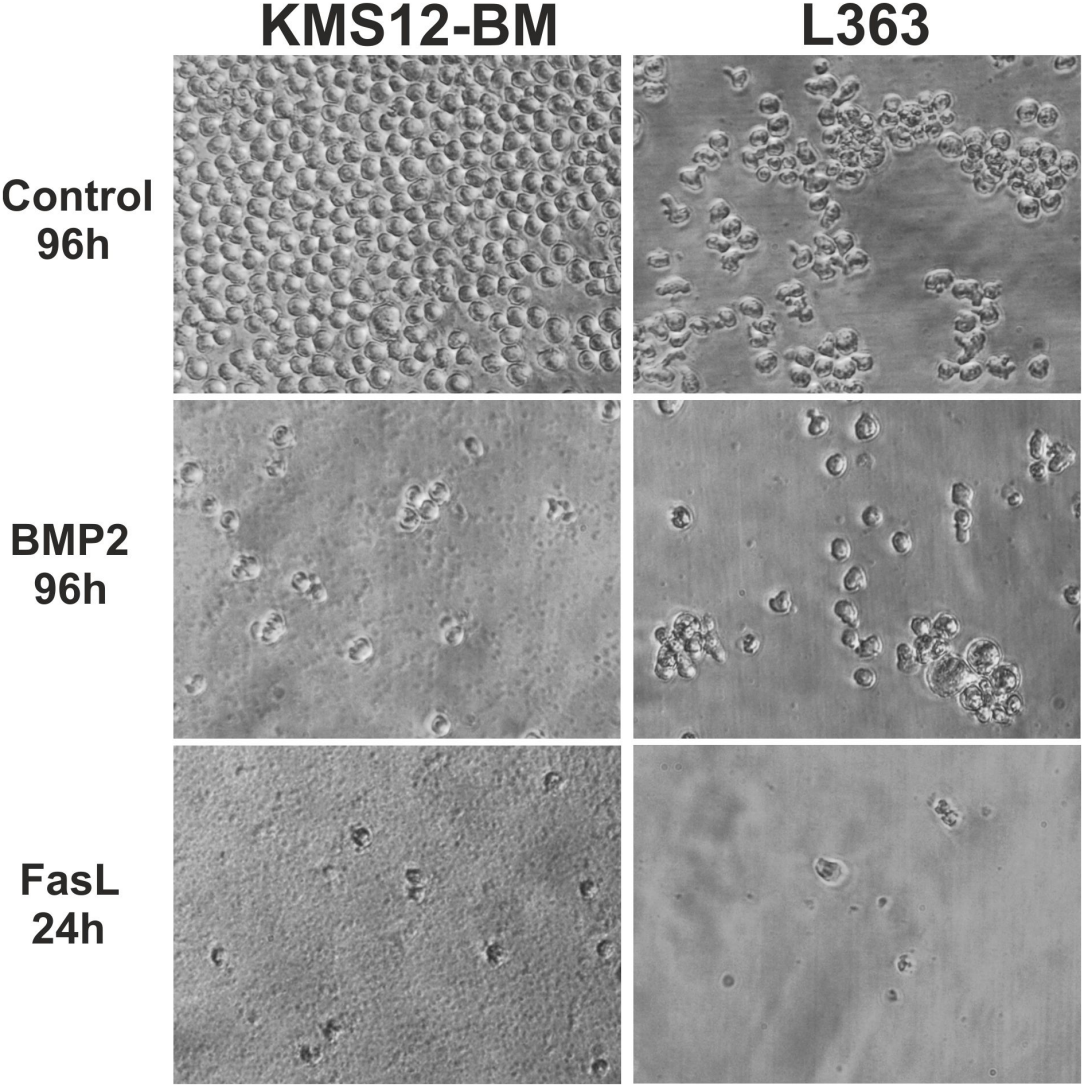
A+B. Microscopic inspection of morphological changes in BMP2- and FasL-stimulated cells. (A) KMS12-BM and (B) L363 cells were incubated in the presence or absence of BMP2 (250 nM) for 96 h. As a positive control for apoptosis, cells were stimulated with FasL (100 ng/ml) for 24 h.

These data suggest that neither apoptosis nor necroptosis contributed significantly to the observed inhibitory effect of BMP2 on cell number and cellular metabolic activity. Because our results suggested that BMP2 exerts only an anti-proliferative effect, we compared the cellular morphology of KMS12-BM and L363 cells upon stimulation with either BMP2 or FasL. FasL is a TNF-related cytokine that was shown to induce apoptosis in these two MM cell lines. After 24 h, visual inspection of FasL-stimulated cells confirmed that the cells were almost completely disintegrated. In contrast, no clear morphological differences could be observed between BMP2-stimulated cells and cells in the untreated controls in either cell line even after 96h, except for differing cell numbers (Fig 5A and 5B).

### Caspase-8 and -3 are not activated upon stimulation with BMP2

**Fig 6:**
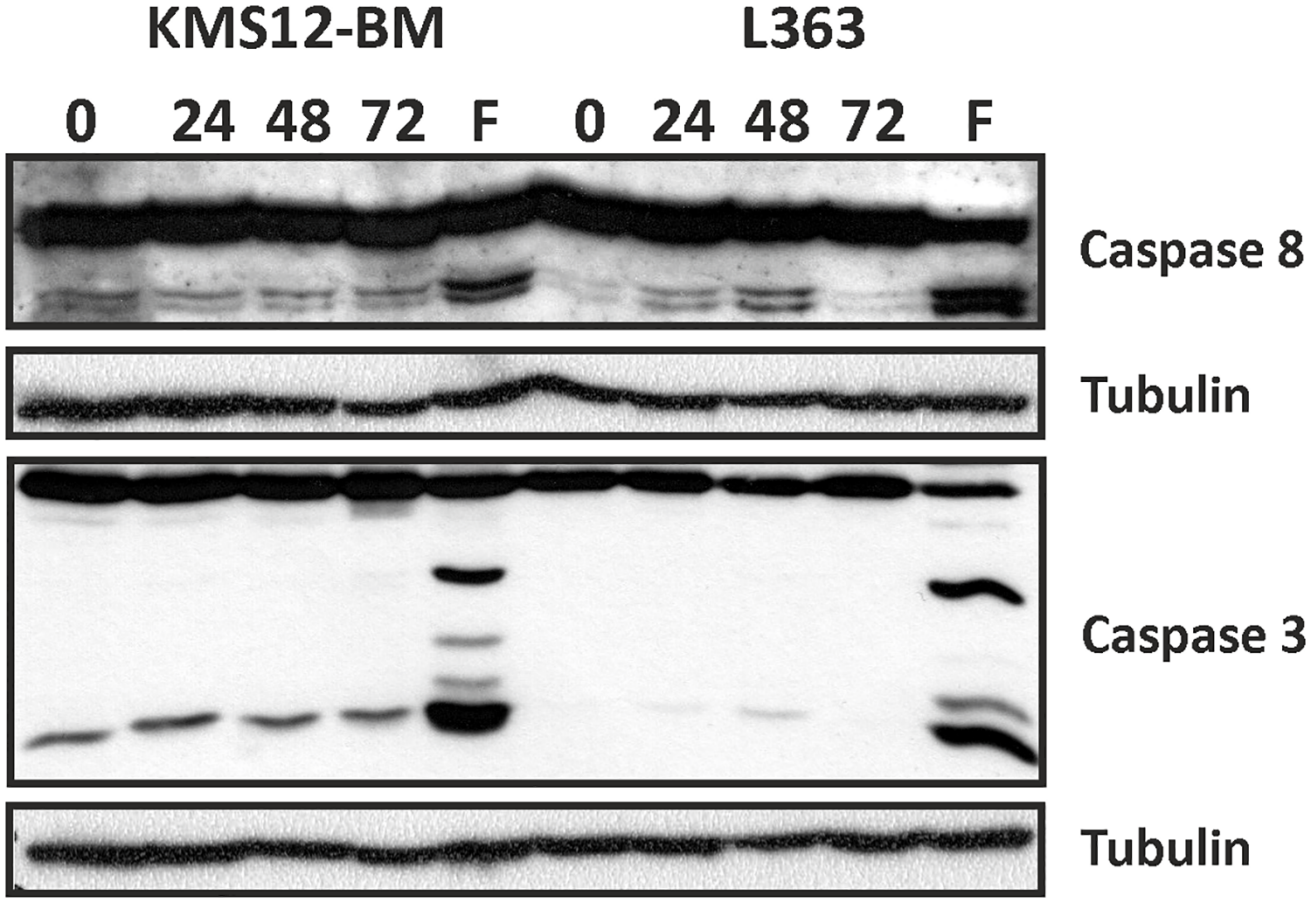
Western blot analysis of caspase 3 and 8 activation. Cells were stimulated for 0 h, 24 h, 48 h and 72 h with BMP2 (250 nM) or for 6 h with FasL (200 ng/ml). Whole cell lysates were separated using SDS-PAGE and proteins were subsequently transferred to nitrocellulose membranes. Immunoblotting was performed using antibodies specific for the proteolytically activated forms of caspase 3 and 8.

In both intrinsic and extrinsic apoptosis pathways, caspases are activated [34, 47, 48]. To corroborate our hypothesis that BMP2 does not induce apoptosis in MM cells we therefore analysed whether BMP2 stimulation resulted in proteolytic activation of the initiator caspase-8 and effector caspase-3 employing Western blot analysis at 0 h, 24 h, 48 h and 72 h stimulation. For positive control, cells were treated with FasL for 6 h. In the lysates of FasL-stimulated cells, we found that both caspases were processed and activated consistent with initiation of apoptosis. In BMP2-stimulated cells, however, both caspases were either not processed at all (L363) or were processed only at very low levels (KMS12-BM) (Fig 6).

### BMP2 does not activate expression of programmed cell death genes

We were puzzled by the fact that the apoptotic effects of the BMPs described in literature were observed only after rather long incubation times (48 h-72 h), which is in marked contrast to the effects of approved apoptotic factors, such as TRAIL or FasL [11–13, 15, 16]. Our results obtained so far clearly indicate that BMP2 does not directly trigger cell death pathways. We therefore sought to determine whether BMP2 activates or inhibits the expression of factors involved in the regulation of programmed cell death in more detail using semi-quantitative real-time PCR and the Qiagen "Cell Death Pathway Finder". This array contains a total of 87 probes specific for genes that play major roles in apoptosis, necroptosis and autophagy (for a list of the included genes, see the Methods section). As time point for the analysis we used 48 h stimulation with BMP2 as at this time point a significant difference in the cell numbers was observed between BMP2-treated and non-treated cells, consistent with the findings reported in the literature [11–13, 15, 16].

**Fig 7:**
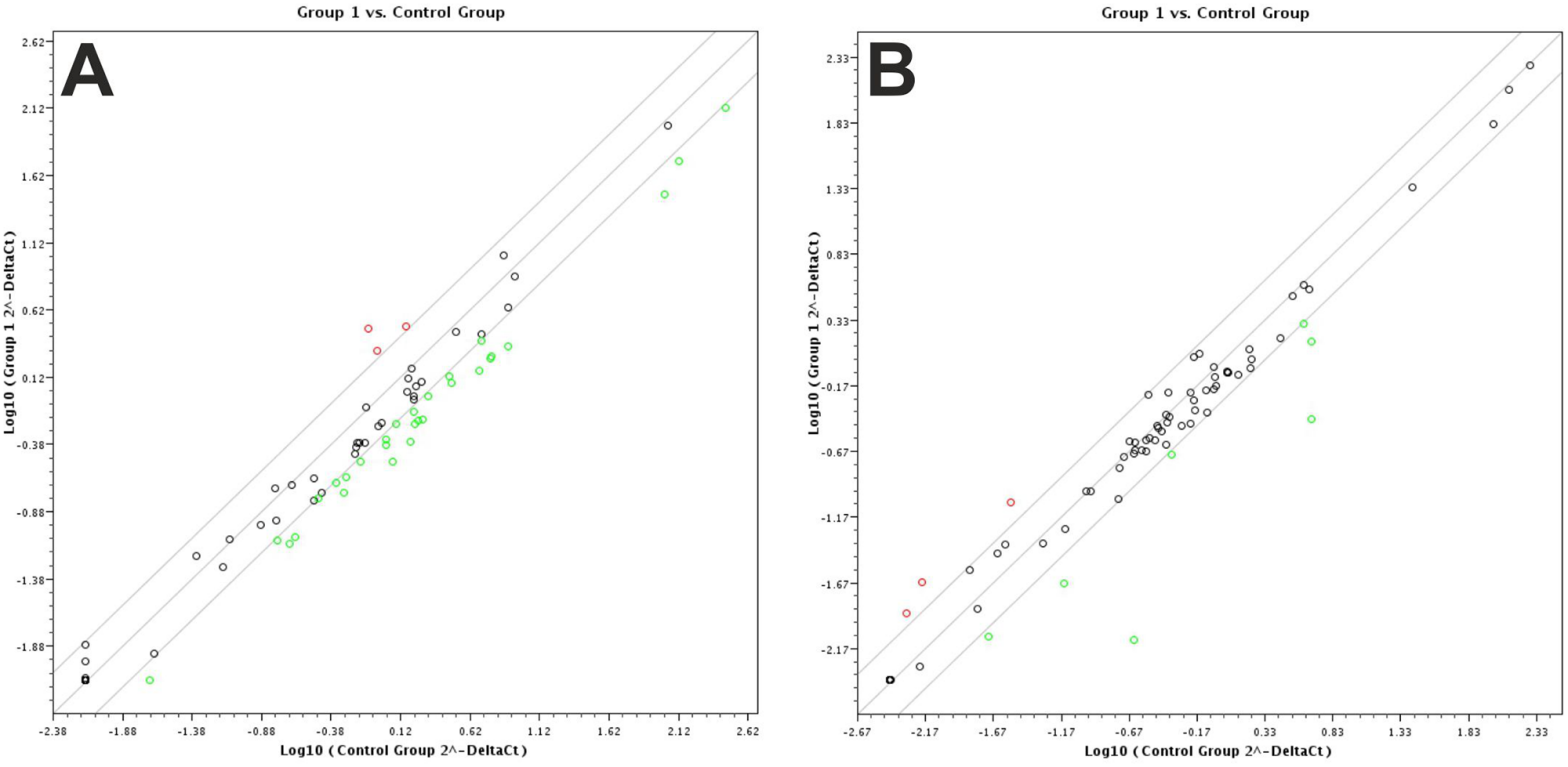
A+B. Expression profiling of cell death pathway genes. (A) KMS12-BM and (B) L363 cells were treated with or without BMP2 (250 nM). RNA was isolated after 48 h. After cDNA synthesis, differential expression was analysed using Real Time RT-PCR and the Qiagen “cell death pathway finder” Array. ΔCt values are indicated as log10 (2^-DeltaCt) values. The scatter plot shows the results correlating the control group against the BMP2-stimulated group. Red circles indicate enhanced gene expression, and green circles indicate decreased gene expression in the BMP2-stimulated group. All differentially expressed genes are listed in Table 1.

At RNA level we found no clear evidence for a regulation of programmed cell death genes by BMP2. The RNA levels of most of the genes examined were either unaltered or down-regulated. The results of our gene expression analysis of both cell lines are shown as scatter plot (Fig 7A and 7B). The degree to which the expression of the analysed genes was changed is shown in comparison to the control. Overall, three genes were up-regulated in both cell lines, while 25 genes were down-regulated in KMS12-BM cells and seven genes were down-regulated in L363 cells. All genes analysed are listed in Table 1. Genes that were up-regulated by 2.3- to 3.9 fold in KMS12-BM (ATG3, BCL2L11 and MCL1) and L363 (BCL2A1, BMF and CASP1) cells were not conclusive as to whether programmed cell death is activated. The large number of down-regulated genes instead suggests that cellular activity was decreased.

**Table 1:** Cell death pathway genes that were differentially expressed upon stimulation with BMP2.

KMS12-BM				L363	
Gene Symbol	X fold	Gene Symbol	X fold	Gene Symbol	X fold
	**UP**			**UP**	
ATG3	3,9			BCL2A1	3,4
BCL2L11	2,2			BMF	3,1
MCL1	2,3			CASP1	2,6
	**DOWN**			**DOWN**	
ATG12	-2,1	GRB2	-2,30	GADD45A	-2,4
ATG16L1	-2,2	HSPBAP1	-2,10	IFNG	-3,4
BAX	-2,0	HTT	-2,20	MAPK8	-29,7
BIRC3	-3,0	IFNG	-2,80	MCL1	-12,5
C1orf159	-2,7	PARP1	-3,20	PARP1	-2,0
CASP1	-2,1	PARP2	-2,50	PARP2	-2,2
CASP2	-2,6	RPS6KB1	-2,30	HPRT1	-3,2
CASP3	-2,9	SPATA2	-2,10		
DENND4A	-2,2	TNFRSF10A	-3,50		
DFFA	-3,2	TNFRSF1A	-2,80		
EIF5B	-3,3	TRAF2	-2,20		
FAS	-3,7	TXNL4B	-2,80		
GADD45A	-2,8				

The expression levels of differentially expressed genes are shown as x-fold values. Positive values indicate an increased expression level, while negative values indicate a decreased expression level in BMP2-stimulated cells.

### BMP2 does not induce apoptosis in primary MM cells

**Fig 8:**
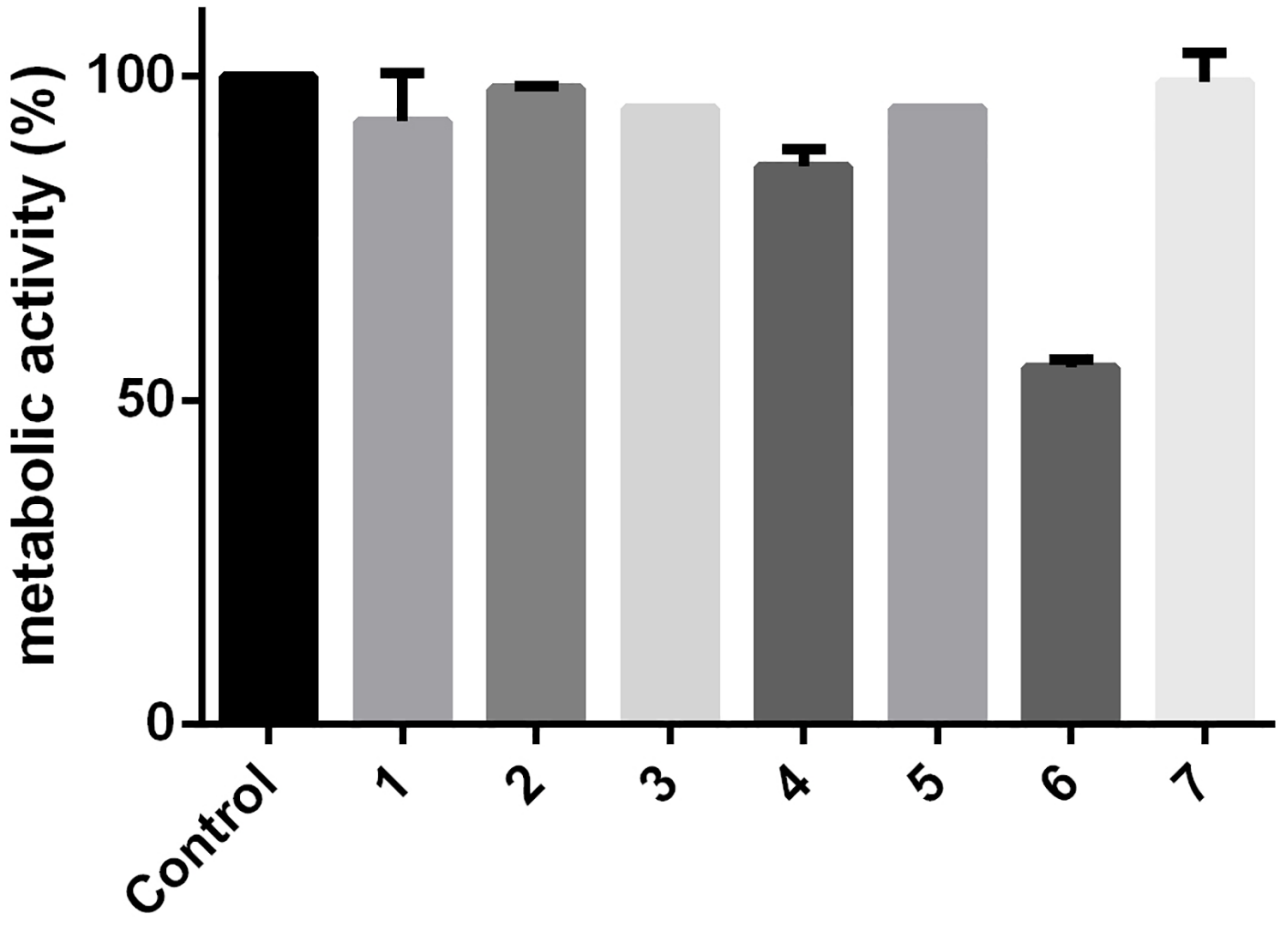
BMP2 has no effect on metabolic activity in CD138+ cells. CD138^+^ cells were seeded at a density of 30,000 cells /well in 96-well plates, and BMP2 was added (250 nM). The cells were then cultured for 3 days after which WST-1 assays were performed. The final values for metabolic activity were calculated relative to those for the untreated control cells. Each assay was performed in duplicate unless a low number of MM cells permitted only single value measurements (no error bar).

To further explore physiological significance of these results, CD138+ plasma cells obtained from human donors with MM were analysed. In total, cells obtained from seven donors were analysed using WST-1 to measure cellular metabolic activity in response to treatment of the cells with BMP2. Only the cells of one donor showed reduced metabolic activity (by 53%) after 72 h stimulation with BMP2. The cells of all other subjects showed no or only marginally decreased cellular activity (Fig 8).

## Discussion

Since the publication of Kawamura *et al*. in 2000, a number of BMPs have been reported to exert apoptotic effects in primary B cells and MM cell lines [11–16]. Experimental methods for detecting apoptosis include MTT assays [11, 14], [3H]-thymidine incorporation [12, 13], the specific degradation of DNA [11, 14] or testing for cell surface expression of Annexin V [12, 14–16]. However, our observations do not corroborate these previously published results. While the anti-proliferative effect of BMP2 could be reproduced, in our experiments BMP2 did not significantly induce programmed cell death, regardless as to whether apoptosis or necroptosis was evaluated. Consequently, BMP2 did not function like a classical apoptotic factor in our experiments, as is found with TRAIL or FasL, and appeared to exert only anti-proliferative activity in the KMS12-BM and L363 human cell lines. This assessment is based on three key facts. First, the time required for BMP2 stimulation to observe the inhibitory effect on cellular viability is too long (i.e., 48 h to 72 h) to be consistent with the fast direct effects exerted by approved apoptotic factors. Second, absolute cell numbers were not or only slightly decreased by BMP2 even after cells have been treated for extended incubation times of up to 4 days. Third, two known inhibitors of programmed cell death, zVAD-fmk and Nec-1, failed to impede the anti-proliferative effect induced by BMP2, while both fully abrogate apoptosis induced by the apoptotic factor FasL.

Apoptosis is fundamentally important to a variety of biological processes, including development (e.g., limb formation [19]) and negative selection in lymphocytes [18]. In addition, apoptosis plays an important role in the daily elimination of potentially malignant or already malignant cells to ensure cellular integrity of the organism. Programmed cell death features two hallmarks: cell death must be exerted fast and it must be highly efficient to avoid residual malignant cells. So-called death ligands found in the TNF family, such as FasL or TRAIL, initiate apoptosis killing a cell within only a few hours. When cells were treated with FasL as a positive control, apoptosis-related events were readily detectable in Western blot analysis after 6 h, and after 24 h, the vast majority of cells were found dead on microscopic examination. By contrast, even after 96 h of stimulation with BMP2, MM cells exhibited no morphological changes, and while the absolute number of cells did not increase in treated cells compared to untreated cells, cell numbers were also not significantly decreased as would be expected from apoptosis. These observations are consistent with the findings described in literature, which report that significant differences between BMP treated and non-treated cells had been observed only after 48 h-72 h by measuring metabolic activity using MTT assays or by quantifying Annexin V expression via flow cytometry [11–13, 15, 16].

Besides the prerequisite of apoptosis to be executed fast, it also must be highly efficient because no *de novo* synthesis of proteins or genetic regulatory events are usually required. Inhibitors of protein synthesis, such as cycloheximide (CHX), can even enhance apoptotic effects [23–26]. Because BMP2 requires more than 48 h to exert its anti-proliferative effect on MM cells, it may however function as an indirect apoptotic factor. We therefore employed gene expression analysis using the "cell death pathway finder" to analyse the gene expression profile of MM cells 48 h after stimulation with BMP2. This allowed us to simultaneously analyse the expression of 87 genes associated with apoptosis, necroptosis and autophagy. However, our analysis convincingly showed that no genes required for activation of programmed cell death were markedly up-regulated. By contrast, numerous genes were down-regulated instead, including genes encoding for anti-apoptotic activity, which strongly suggests that solely cellular activity is reduced. It is well documented that in MM, plasma cells undergo cell-cycle arrest following stimulation with BMP [11, 12, 16]. For instance, Kawamura et al. showed that BMP2 can induce a G1 cell cycle arrest in MM cells [11]. They also concluded that BMP2 first induces cell cycle arrest resulting in an anti-proliferation phase, which is followed by apoptosis. To validate their results they also analyzed the expression of cell cycle-specific genes such as p15, p21, p27, CDK2, CDK4, the cyclins D1-D3, Cyclin E and also studied the phosphorylation level of Rb (retinoblastoma protein) employing western blot analysis. All these genes play an important role in the regulation of proliferation and/or their expression marks the transition to the different cell cycle phases. The two G1-checkpoint CDK inhibitors p21 and p27 showed an increased expression in the time period from 4h to 48h past stimulation with BMP2. Analysis of the phosphorylation status of Rb revealed that 24 h after BMP2-stimulation levels of hyper phosphorylated ppRb were decreased, while levels of pRB were increased. Levels of CDK2, CDK4, cyclin D2, and cyclin E remained unchanged upon BMP2 treatment. In contrast, expression level of cyclin D3 increased after 4h and gene expression level of cyclin D1 were decreased after 48h exposure to BMP2. Further studies of Kawamura et al. have confirmed that Activin A as well as BMP2, both members of the TGFbeta-family, induce cell cycle arrest in the G1 phase [11, 33]. While p27 also seems to be important, the regulation was different in human and mice. Fukuda et al. showed that expression of p53, which plays a key role in cell cycle transition regulation, is increased upon BMP4 stimulation [14]. p53 seems indeed a good candidate for regulation by BMP as this protein is not a per-se an apoptotic factor, but functions as tumor suppressor gene. It initiates a proliferation stop by arresting the cell cycle under different conditions to enable a check of the cell viability status. If this check identifies critical parameters potentially impairing proper cellular function the cell will self-destruct, and undergo apoptosis to prevent further damage to the surrounding environment. So by activating p53 BMPs are not real apoptotic factors but act anti-proliferative possibly as a first step towards apoptotic elimination. The observation that some cells undergo cell death might therefore be a consequence of the p53-controlled quality check.

The hypothesis that MM cells are cell cycle arrested by BMPs is fully consistent with the observations in our experiments. Analysis of the absolute cell numbers, which were determined by direct cell counting using a Coulter counter, showed that the number of cells initially subjected into the experiment did not increase significantly during the time course of the experiment, which is due to BMP2 efficiently slowing down cell growth. This effect became most apparent when comparing the cell number of the BMP2-treated group to the numbers of cells in the untreated group, which showed the high proliferation activity of MM cells under non-stimulated conditions. Finally, the failure of two known inhibitors of programmed cell death to rescue BMP2-inhibited synthesis of NAD(P)H, which is the key parameter in assays, such as the MTT (WST1) etc., determining metabolic activity, not only points towards the pitfalls of these assays when applied without further controls [49], but also strongly indicate that BMP2 does not induce the apoptotic elimination of these cells but only stops their further proliferation. This conclusion is further corroborated by a direct comparison of BMP2 with FasL, an approved factor robustly triggering programmed cell death in the myeloma cell lines highlighting the differences in the effects induced by an apoptotic factor and those observed with BMP2.

How can our results be reconciled with evidence in the literature proposing that BMP induces apoptosis? We think that the previous interpretation of BMP2 induces cell death might be due to an over-interpretation of the inhibitory effect of BMP2 on MM cell line growth likely due to two reasons. First, no comparative analysis of the BMP2 action on human MM cells with an approved apoptosis-inducing positive control such as FasL were done, which would have hinted towards mechanistic differences in the action of both growth factors, which is elimination of cells leading to a decrease in cell numbers versus keeping the cell numbers constant due to a proliferation stop. Secondly, the rather long experiment times required to reveal the BMP2 inhibitory effect did not raise concerns that this would violate a key feature of apoptosis, which is its fast and highly efficient execution. The minor signs of apoptosis that have been detected in BMP-treated MM cells are therefore very likely a secondary result of the well-documented process of BMP-induced cell cycle arrest [11, 12, 14, 16], which is caused by a p53-related control mechanism and if maintained over a long period of time can lead to cell death [14, 48].

## Conclusions

In our experiments, BMP2 did not meet the key criteria required to function as an apoptotic factor. Firstly, the time required to exert its activity is too long (i.e. 48 h-72 h), since apoptotic factors generally act within hours. Secondly, the effects induced by known apoptotic factor FasL in MM cells (e.g. change in cell morphology, decrease in absolute cell number due to cell elimination), clearly differs from the effects caused by BMP2. Signs for BMP2-induced apoptosis were only marginal. Thirdly, two approved inhibitors of programmed cell death, zVAD-fmk and Nec-1, did not affect BMP2 activity while cancelling the apoptosis induced by FasL. Based on these findings, we conclude that BMP2 exerts solely an anti-proliferative effect on human neoplastic B cell lines in MM and BMP2-induced cell death only found in a very small subset of the cells is a side-effect of the p53-mediated quality check during cell cycle arrest.
